# Task shifting of emergency caesarean section in south Ethiopia: are we repeating the brain drain

**DOI:** 10.11604/pamj.2020.36.145.19330

**Published:** 2020-07-01

**Authors:** Anteneh Asefa, Alison Morgan, Tadesse Hailemariam, Mekonnen Shiferaw, Emebet Mekonnen, Yifru Birhan

**Affiliations:** 1 School of Public Health, College of Medicine and Health Sciences, Hawassa University, Hawassa, Ethiopia,; 2 Nossal Institute for Global Health, Melbourne School of Population and Global Health, University of Melbourne, Melbourne, Australia,; 3 United Nations Population Fund, SNNPR Office, Hawassa, Ethiopia,; 4 Southern Nations Nationalities and Peoples Regional Health Bureau, Hawassa, Ethiopia,; 5 Federal Ministry of Health, Addis Ababa, Ethiopia

**Keywords:** Attrition, caesarean section, delivery, Ethiopia, primary hospital, task shifting

## Abstract

**Introduction:**

preventable mortality from complications which arise during pregnancy and childbirth continue to claim more than a quarter of million women´s lives every year, almost all in low- and middle-income countries. However, lifesaving emergency obstetric services, including caesarean section (CS), significantly contribute to prevention of maternal and newborn mortality and morbidity. Between 2009 and 2013, a task shifting intervention to train caesarean section (CS) teams involving 41 CS surgeons, 35 anesthetic nurses and 36 scrub nurses was implemented in 13 hospitals in southern Ethiopia. We report on the attrition rate of those upskilled to provide CS with a focus on the medium-term outcomes and the challenges encountered.

**Methods:**

a cross-sectional study involving surveys of focal persons and a facility staff audit supplemented with a review of secondary data was conducted in thirteen hospitals. Mean differences were computed to appreciate the difference between numbers of CSs conducted for the six months before and after task shifting commenced.

**Results:**

from the trained 112 professionals, only 52 (46.4%) were available for carrying out CS in the hospitals. CS surgeons (65.9%) and nurse anesthetists (71.4%) are more likely to have left as compared to scrub nurses (22.2%). Despite the loss of trained staff, there was an increase in the number of CSs performed after the task shifting (mean difference=43.8; 95% CI: 18.3-69.4; p=0.003).

**Conclusion:**

our study, one of the first to assess the medium-term effects of task shifting highlights the risk of ongoing attrition of well-trained staff and the need to reassess strategies for staff retention.

## Introduction

Although global maternal mortality declined by 44% between 1990 and 2015, geographical disparities of maternal mortality widened during the same 25-year period [1]. Preventable mortality from complications which arise during pregnancy and childbirth continue to claim more than 250,000 women´s lives every year, almost all in low and middle-income countries (LMICs) [1]. In Ethiopia, maternal mortality has been significantly reduced from 1,400 per 100,000 live births in 1990 to 412 per 100,000 live births in 2015 [2]. To reach the target within the third sustainable development goal (SDG3) of a MMR less than 140 by 2030, the country is prioritizing both the access and quality of maternal health services [3]. Health systems strengthening and improving access to lifesaving maternal and child health services remains one of the strategic priorities in addressing SDG3 [4]. In LMICs, improving access to comprehensive emergency obstetric and newborn care (CEmONC) and in particular caesarean sections (CS) is one among several interventions to reduce maternal and newborn mortality [5]. The availability of CS within a facility is also associated with more women accessing skilled birth attendance [6]. However, limited access to services and shortage of skilled personnel are still deterrents to expanding the availability of CS services [7]. Globally, there is a big disparity in rates of CS; rising CS rate due to unjustifiable CSs is becoming a concern, especially in middle and high-income countries [5, 8].

In contrast, in sub-Saharan Africa, CS rates increased from 2.3% to just 3.5% between 1990 and 2014 [8]. The rate of CS was particularly low (1.9%) in Ethiopia and the disparity in access exemplified with the high rate (21.4%) in the capital, Addis Ababa [9]. CS requires health workers skilled in anesthetics, surgery and a facility able to provide the requisite nursing care and the capacity to provide blood transfusion services. In Ethiopia in 2015, there were only 174 obstetricians and gynecologists working in public health hospitals and administrative organizations [10]. In addition, just 18% of the population live <80km from a CEmONC facility [11]. Consequently, task shifting, a process whereby specific tasks are moved, where appropriate, to health workers with shorter training and fewer qualifications [12], has been an approach to improve the accessibility of CS. Similar approaches have been effective in sub-Saharan African and south-east Asian countries without posing significant adverse outcomes [13]. Previous research in Ethiopia reported that shifting CS tasks to non-physician clinicians improved access and yielded equivalent outcomes to obstetrician provided CS [14]. This study reports on the attrition amongst teams of personnel trained to conduct CS in 13 primary level hospitals (where obstetricians and gynecologists were either not available or scarce) in southern Ethiopia. The task shifting was part of a comprehensive program to improve the accessibility to CEmONC services.

## Methods

**Description of task shifting intervention:** the task shifting of CS was implemented in 13 primary hospitals (Adare, Bona, Bonga, Chencha, Durame, Gidole, Halaba, Karat, Kelle, Leku, Sawula, Tercha and Worabe) in southern nations nationalities and peoples region (SNNPR) between 2010 and 2013. All study hospitals are located in different administrative zones or districts of the region. To fully run CS in the primary hospitals, the following interventions were done: three months intensive training of CS teams by obstetricians and gynecologists; equipping hospitals with necessary logistics and commodities; and supportive supervision and mentoring after deployment of trained professionals. Every training team comprised of either a general practitioner (GP) or health officer (HO) trained as a CS surgeon, a nurse trained as a scrub nurse and a nurse trained as an anesthetist nurse. The CS surgeon is responsible for leading the entire team and performing the surgery whereas the anesthetist nurse administers the spinal anesthesia and monitors vital signs; scrub nurses ensure availability and completeness of all tools before and after a surgery, transfer patients and assist surgeons during CS procedures. In Ethiopia, GPs and HOs are not trained to conduct CS in their regular studies. Similarly, the clinical nurses who were trained to be anesthetist nurse and scrub nurse in the task shifting were not initially trained to perform these tasks in their regular studies. A total of 41 GP/HO surgeons, 35 anesthetic nurses and 36 scrub nurses were trained (Table 1).

**Table 1:** summary of health professionals trained on CEmONC and availability of CS and blood transfusion services

Hospitals	CEmONC Trainees by category (n)					Availability of CS			Availability of blood transfusion		
	GP (A)	HO (B)	Surgeons (A+B)	Anesthetic Nurse	Scrub Nurse	Continuous^*^	Intermittent^**^	Never	Continuous	Intermittent	Never
Adare Hospital	2	1	3	2	2	✓			✓		
Bona Hospital	1	0	1	1	1		✓			✓	
Bonga Hospital	2	4	6	5	6	✓				✓	
Chencha Hospital	1	2	3	4	1	✓				✓	
Durame Hospital	2	0	2	2	2	✓			✓		
Gidole Hospital	0	3	3	3	2	✓				✓	
Halaba Hospital	3	1	4	4	4		✓			✓	
Karat Hospital	0	3	3	2	2	✓				✓	
Kelle Hospital	0	1	1	1	1		✓				✓
Leku Hospital	3	0	3	3	3		✓			✓	
Sawula Hospital	1	1	2	2	2	✓				✓	
Tercha Hospital	6	3	9	6	4	✓				✓	
Worabe Hospital	1	0	1	0	6	✓					
Total trained	22	19	41	35	36						
Currently available	6	8	14	10	28						
**Reason for attrition; n (%)**											
Resignation	3 (18.8)	4 (36.4)	7 (25.9)	1 (4)	1 (12.5)						
Career education	10 (62.5)	4 (36.4)	14 (51.9)	9 (36)	3 (37.5%)						
Transfer	2 (12.5)	3 (27.3)	5 (18.5)	4 (16)	1 (12.5)						
Rotation	1 (6.3)	-	1 (3.7)	11 (44)	3 (37.5%)						
Total attrition	16	11	27	25	8						

* through blood bank; ^**^through family members or list of blood donors for district hospitals, only through list of blood donors for regional hospital

**Study design:** this study employed a cross-sectional design and was conducted between January and March 2015. Surveys of maternity unit focal persons and review of secondary data were done in all study hospitals. Secondary data were extracted from labor, delivery and CS registers. Two days of intensive training on the data collection tools followed by pretesting of the tools was offered to two data collectors before data collection. A questionnaire was administered to the maternity unit focal persons to generate information about the number of currently available CS trained professionals and the reasons for their unavailability, in cases where one or more professionals were found to be inactive in the CS service delivery. Our study extracted the number of CSs performed during the six months preceding the deployment of CS trained teams and compared this with the number of CSs performed during the six months after deployment of trained teams. Furthermore, disaggregation of CSs was made to assess the proportion of CSs conducted by GP/HO surgeons. The index date to classify events of CSs in the hospitals was based on the first date when the first cohort of CS trained teams, mainly the first trained surgeon, started to provide CS services. In four of the hospitals (Bonga, Durame, Gidole and Tercha), the index date was December 2011. The index dates for the remaining hospitals vary (Table 2).

**Table 2:** comparison of performance of caesarean section and delivery before and after task shifting in the study hospitals

Hospitals	Index date of task-shifting	#of CS 6m before task shifting (n)	#of CS 6m after task shifting (n)		Proportion by GP/HO surgeon (C/B)*100	Absolute change (percent change)	Delivery		
			Overall (B)	By GP/HO surgeon(C)			#6m before task shifting	#6m after task shifting	Absolute change (Percent change)
Bona Hospital	March 2012	37	49	18	36.7%	12 (+32%)	258	354	96 (+37.2%)
Bonga Hospital	December 2011	22	26	20	76.9%	4 (+18%)	NAv	NAv	-
Chencha Hospital	August 2013	0	32	8	25.0%	32 (NA)	307	340	33 (+10.7%)
Durame Hospital	December 2011	136	138	66	47.8%	2 (+1%)	476	514	38 (+8.0%)
Gidole Hospital	December 2011	15	67	30	44.8%	52 (+347%)	193	388	195 (+101.0%)
Halaba Hospital	June 2013	0	117	60	51.3%	117 (NA)	196	565	369 (+188.3%)
Karat Hospital	April 2013	22	74	0	0.0%	52 (+236%)	148	529	381 (+257.4%)
Kelle Hospital	September 2013	0	5	0	0.0%	5 (NA)	NAv	NAv	-
Leku Hospital	October 2013	0	61	16	26.2%	61 (NA)	202	361	159 (+78.7%)
Sawula Hospital	February 2013	51	131	49	37.4%	80 (+157%)	366	506	140 (+38.3%)
Tercha Hospital	December 2011	11	18	18	100.0%	7 (+64%)	91	83	83 (-8.8%)
Worabe Hospital	December 2013	0	102	21	20.6%	102 (NA)	NAv	NAv	-
Total		294	820	306	37.3%	526 (+179%)	2237	3640	1403 (+88%)
Mean		24.5	68.3	25.5					

**Data analysis and presentation:** data was entered into and analyzed using SPSS version 20 (IBM, Armonk, NY, USA) software. Description of the number of trained CS team and their availability/unavailability for conducting CS services is made. Additionally, the number of CSs conducted and deliveries attended during the six months before and after index date of the task shifting was presented for every hospital. As displayed in Table 2, that resulted in12 different pre and post-intervention observations. In Adare Hospital, the CS registers used during the study´s target period were misplaced and data could not be extracted. Paired samples T-test was done to check for difference between the mean number of CSs and total deliveries before and after the task shifting.

**Ethics and consent:** this study received an ethical approval from the institutional review board located in SNNPR Health Bureau, Ethiopia. Additionally, permission letters were granted from SNNPR Health Bureau and the hospitals included in the study. Verbal consent was given by all CEmONC focal persons included in the study.

## Results

Among the focal persons (n=13) surveyed, four were nurses, while the remaining were integrated emergency surgical officers (3), midwives (2), HOs (2) and physicians (2). Focal persons´ mean service year in their respective hospitals was 2.42±1.98 years; whereas as serving as a focal person was 1.59 ± 1.27 years. From the total number of trained professionals (112), only 52 (6 GPs, 8 HOs, 10 anesthetist nurses and 28 scrub nurses) were active on-site (available for carrying out CS procedure in the facility; not necessarily present on the date the site visit was made). This means only 27.3% of GPs, 42.1% of HOs, 28.6% of anesthetist nurses and 77.8% of scrub nurses were retained in CS service delivery departments/units of the hospitals. In Tercha Hospital, the problem is worse; of 9 CS surgeons, none were active on duty. Reported reasons for non-retention of CS trained providers were staff transfer, study leave for career education and resignation were the major ones. Study leave for career education was the most reported reason for non-retention of CS surgeons whereas it was resignation for anesthetist nurses (Table 1). During the six months preceding initiation of task shifting, CS was not available in four (Chencha, Halaba, Kelle and Leku) of the twelve hospitals included in the analysis. Additionally, from the total of CSs conducted in Karat (74) and Kelle (5) hospitals after the task shifting process, none of the CSs were conducted by GP/HO surgeons.

Reason/s for those experiences were reported to be the absence of blood transfusion service and fear of power interruption amid CS procedure in Kelle Hospital and new deployment of an overseas supported surgeon in Karat Hospital (Table 2). Generally, the number of CSs conducted increased in all hospitals after the task shifting process; overall, the number of CS increased by 179%. This has been evidenced by a significant increment after the task shifting (mean difference = 43.8: 95% CI = 18.3, 69.4; p = 0.003) (Table 3). The number of CSs conducted more than doubled in Gidole, Karat and Sawula hospitals. No correlation was found between individual hospitals pre-task shifting and post-task shifting performance of CS (p = 0.06) (Table 3). This lack of correlation compounded by small sample size to control the effects of potential confounders make impossible attributing the improved performance of CSs to the task shifting. Apart from this, there was also significant difference between the total number of deliveries attended between the pre and post task shifting periods (Table 3). CS and blood transfusion services are the core elements of CEmONC. Hence, their dual availability in hospitals facilitate delivery of high-quality lifesaving interventions. However, in the current study, blood transfusion service was continuously available in only two of the hospitals in one-year period preceding the survey; this might have impeded the hospitals from rendering CS service at some point in time in the past. On the other hand, CS was rendered continuously in 9 of the study hospitals; it was intermittently available in the remaining 4 hospitals (Table 1).

**Table 3:** statistical test of significance for difference between performance of caesarean section and delivery before and after task shifting

Activities in all hospitals	6 months before task shifting		6 months after task shifting		Mean Difference (95% CI)	T	n	df
	μ	σ^2^	Μ	σ^2^				
Caesarean sections performed	24.5	38.8	68.3	45.1	43.8 (18.3, 69.4)	3.78^*^	12	11
Total deliveries attended	248.6	118.3	404.4	148.2	155.9 (48.9, 263.7)	3.34^**^	9	8

μ=mean, σ2=standard deviation, df=degree of freedom; ^*^p=0.003 ^**^p=0.02

## Discussion

Our study shows that there was high attrition (46.4%) of the personnel trained for CS task-shifting within 13-15 months of deployment at the study hospitals. The attrition was higher among CS surgeons (65.9%) and anesthetist nurses (71.4%) as compared scrub nurses (22.2%). Study leave for career education and resignation were the commonest reasons for attrition. We believe that high attrition casts a dark shadow on the cost-effectiveness and sustainability of the intervention if adequate preparation of retaining trained staff is not undertaken in advance. Similar challenges of retaining CS surgeons have been reported by other task shifting processes carried out in Senegal, Uganda and India [15]. Retaining surgeons in rural catchments is a multifaceted challenge and possible solutions start with dealing with the negative impact of their job on lifestyle [16]. Challenges in retaining anesthetic trained personnel was also experienced by other several south Asian countries [17]. According to the World Health Organization, adopting appropriate staff retention mechanism is key to ensuring the sustainability of task shifting and to address challenges confronting health systems in LMICs [12, 18]. According to one commentary, many retention programs fail, ostensibly because they are aimed at preventing or restricting health care employees from leaving and are not really aimed at addressing the factors that keep them on the job [19].

Our study provides supporting evidence that the same challenges that prevent obstetricians from working in remote settings are likely to apply to GPs and HOs trained to perform similar functions. Task shifting is a strategy to address health workforce shortages. Immediate increases in service utilization as observed in this study are possible. Yet the attrition of professionals to provide the services they were meant to was also observed and just 52 of the 112 staff trained to provide CS services were available. Task shifting of CS services cannot be an end by itself in making the service more accessible; there are many health systems issues intercepting its implementation. Lack of decision making power at district health system, delay in financial disbursements [20] and undefined roles and perceived risk of the task shifting by senior clinicians [18] are some of the challenges articulated. A qualitative study from Uganda also described the lack of policy and guidelines on task shifting and poor planning as barriers to effective task shifting [21]. A significant increase in the numbers of CSs performed and total deliveries attended was observed within six months after the task shifting. Attributing the increments solely to the task shifting is not appropriate due to various reasons mainly: ongoing improvement in infrastructural readiness of the hospitals, a general increase in the demand for facility-based childbirth over time and ongoing strengthening of the referral linkages between health facilities. A similar increase in the number of CSs conducted after task shifting of CSs was documented in north Ethiopia [22].

In the current study, CS and blood transfusion services were continuously available in 9 (69.2%) and 2 (15.4%) of the study hospitals respectively 13-15 months after the intervention. A study from Burkina Faso revealed comparable levels of continuous availability CS (53.5%) and blood transfusion (20.9%) [23]. Shortage of blood transfusion facilities might have posed health facilities to refer women to next referral pathways due to fear of blood loss; though this is arguable by most scholars in the field. Studies in different countries and different levels of hospitals reported varying rates of blood transfusion during CS procedures; Australia: 0.63% [24], Malawi: 7.2% [25] and Nigeria: 25.2% [26]. Therefore, building the capacity of district health systems in availing blood transfusion facilities is of pivotal importance to improve access and coverage of CS through task shifting. Lastly, this study has several limitations inherent to the design used. First, pre and post measurements are prone to events which might have happened concurrent to task shifting implementation. Furthermore, analysis of six months aggregate data before and after task shifting initiation made it impossible to appreciate the trend of CSs overtime. The difference in index dates of task shifting poses an additional challenge as the level and intensity of interventions/supports may vary across time. Furthermore, as a stand-alone quantitative design dependent on data from secondary sources, this study is prone to conventional limitations which arise from incompleteness and inconsistency of data. Due to limited medical record keeping, the current study was not able to include a measurement of CS complications to assess whether the quality of CS differed between different cadres of CS providers. Therefore, we recommend future studies use a longitudinal design to track changes across time to better estimate the effects of task shifting interventions.

## Conclusion

We believe that task shifting of CS still significantly contributes in improving the accessibility of CS services in rural Ethiopia. However, more attention is required to provide supportive environment to retain newly trained CS teams in rural settings, as there is a risk that these professionals will become as difficult to retain as the health providers they are designed to replace.

### What is known about this topic

- Improving access to CS is one among several interventions to reduce maternal and newborn mortality;
- Evidence shows that CS can be successfully task-shifted to non-specialist physicians and non-physicians in low income settings.

### What this study adds

- Ongoing attrition of CS trained professionals was found to be a challenge in ensuring continuous operation of CS in the study hospitals;
- Reassessing the strategies for staff retention should be done.

## References

[1] World Health Organization (2015). Trends in maternal mortality: 1990 to 2015: estimates by WHO, UNICEF, UNFPA, World Bank Group and the United Nations Population Division.

[2] Central Statistical Agency (2017). Ethiopia demographic and health survey 2016. Central Statistical Agency.

[3] Ministry of Health (2015). Health sector transformation plan 2015/16-2019/20.

[4] Shetty AK (2016). Global maternal, newborn and child health: successes, challenges and opportunities. Pediatr Clin North Am.

[5] World Health Organization (2015). WHO Statement on caesarean section rates. Reprod Health Matters.

[6] Stanton C, Blanc AK, Croft T, Choi Y (2007). Skilled care at birth in the developing world: progress to date and strategies for expanding coverage. Journal of biosocial science.

[7] Irani M, Deering S (2015). Challenges affecting access to cesarean delivery and strategies to overcome them in low-income countries. Int J Gynaecol Obstet.

[8] Betran AP, Ye J, Moller AB, Zhang J, Gulmezoglu AM, Torloni MR (2016). The increasing trend in caesarean section rates: global, regional and national estimates: 1990-2014. PLoS One.

[9] Gebremedhin S (2014). Trend and socio-demographic differentials of caesarean section rate in Addis Ababa, Ethiopia: analysis based on Ethiopia demographic and health surveys data. Reprod Health.

[10] Ministry of Health (2016). Human resource for health in Ethiopia.

[11] McKinnon B, Harper S, Kaufman JS, Abdullah M (2014). Distance to emergency obstetric services and early neonatal mortality in Ethiopia. Trop Med Int Health.

[12] World Health Organization (2008). Task shifting: global recommendations and guidelines.

[13] Bergstrom S (2015). Training non-physician mid-level providers of care (associate clinicians) to perform caesarean sections in low-income countries. Best Pract Res Clin Obstet Gynaecol.

[14] Gessessew A, Barnabas GA, Prata N, Weidert K (2013). Task-shifting in obstetric care. Int J Gynaecol Obstet.

[15] Schneeberger C, Mathai M (2015). Emergency obstetric care: making the impossible possible through task shifting. Int J Gynaecol Obstet.

[16] Cogbill TH, Cofer JB, Jarman BT (2012). Contemporary issues in rural surgery. Curr Probl Surg.

[17] Mavalankar D, Sriram V (2009). Provision of anaesthesia services for emergency obstetric care through task shifting in South Asia. Reprod Health Matters.

[18] Deller B, Tripathi V, Stender S, Otolorin E, Johnson P, Carr C (2015). Task shifting in maternal and newborn health care: key components from policy to implementation. Int J Gynaecol Obstet.

[19] Rondeau KV (2002). Health human resource planning requires an effective staff retention policy. Healthc Pap.

[20] Frumence G, Nyamhanga T, Mwangu M, Hurtig AK (2014). The dependency on central government funding of decentralised health systems: experiences of the challenges and coping strategies in the Kongwa District, Tanzania. BMC Health Serv Res.

[21] Dambisya YM, Matinhure S (2012). Policy and programmatic implications of task shifting in Uganda: a case study. BMC Health Serv Res.

[22] Gessessew A, Barnabas GA, Prata N, Weidert K (2011). Task shifting and sharing in Tigray, Ethiopia, to achieve comprehensive emergency obstetric care. International Journal of Gynaecology and Obstetrics.

[23] Compaore GD, Sombie I, Ganaba R, Hounton S, Meda N, Brouwere VD (2014). Readiness of district and regional hospitals in Burkina Faso to provide caesarean section and blood transfusion services: a cross-sectional study. BMC Pregnancy Childbirth.

[24] Chua SC, Joung SJ, Aziz R (2009). Incidence and risk factors predicting blood transfusion in caesarean section. Aust N Z J Obstet Gynaecol.

[25] Fenton PM (1999). Blood transfusion for Caesarean section in Malawi: a study of requirements, amount given and effect on mortality. Anaesthesia.

[26] Ozumba BC, Ezegwui HU (2006). Blood transfusion and caesarean section in a developing country. J Obstet Gynaecol.

